# Dormancy and germination of microsclerotia of *Verticillium longisporum* are regulated by soil bacteria and soil moisture levels but not by nutrients

**DOI:** 10.3389/fmicb.2022.979218

**Published:** 2022-09-23

**Authors:** Sarenqimuge Sarenqimuge, Shahinoor Rahman, Yao Wang, Andreas von Tiedemann

**Affiliations:** ^1^Plant Pathology and Plant Protection Division, Department of Crop Sciences, Faculty of Agriculture, Georg-August University Göttingen, Göttingen, Germany; ^2^Agricultural Entomology Division, Department of Crop Sciences, Faculty of Agriculture, Georg-August University Göttingen, Göttingen, Germany

**Keywords:** soil bacteria, soil fungistasis, bacterial volatiles, *Brassica napus*, GC–MS

## Abstract

The soil-borne pathogen *Verticillium longisporum* infects roots of its host plant, oilseed rape, and systemically colonizes stems where it finally forms microsclerotia at crop maturity. Once returned to the soil after harvest, microsclerotia undergo a stage of dormancy, in which they may survive for several years. Since there is neither efficient chemical control nor effective resistance in oilseed rape cultivars to control the disease, alternative control strategies may consist in regulating the germination and dormancy of microsclerotia in the soil. Therefore, a series of experiments were conducted to explore the effects of nutrients, soil moisture, and the soil microbiome on germination of dormant microsclerotia. Experiments with microsclerotia exposed *in vitro* to different nutrients indicated that under sterile conditions the stimulating effect of nutrients on microsclerotia germination was not enhanced as compared to water. Moreover, further assays revealed a strong inhibitory effect of unsterile soil on microsclerotia germination. Accordingly, oilseed rape plants inoculated with microsclerotia of *V. longisporum* showed severe infection with *V. longisporum* when grown in autoclaved soil, in contrast to plants grown in unsterile soil. These experiments indicate a crucial role of soil fungistasis and thus the soil microbiome on microsclerotia germination. Further bioassays demonstrated that viable soil bacteria obtained from the rhizosphere of oilseed rape plants and bulk field soil effectively inhibited microsclerotia germination, whereas dead bacteria and bacterial culture filtrates hardly suppressed germination. A putative inhibitory role of volatile organic compounds (VOCs) produced by soil bacteria was confirmed in two-compartment Petri dishes, where microsclerotia germination and colony growth were significantly inhibited. Bacterial VOCs were collected and analyzed by GC–MS. In total, 45 VOCs were identified, among which two acid and two alcohol compounds were emitted by all tested bacteria. A bioassay, conducted with corresponding pure chemicals in two-compartment Petri dishes, indicated that all acidic volatile compounds, including 3-methylbutanoic acid, 2-methylbutanoic acid, hexanoic acid, and 2-methylpropionic acid, induced strong inhibitory effects on microsclerotia. We conclude that bacterial acidic volatiles play a key role in the fungistatic effect on microsclerotia of *V. longisporum* in the soil and could thus be targeted for development of novel strategies to control this pathogen by artificially regulating dormancy of microsclerotia in soil.

## Introduction

*Verticillium longisporum* is a soil-borne, root infecting and xylem invading pathogen which causes stem striping disease in the economically important crop oilseed rape (*Brassica napus*), a disease which so far occurred in China, Europe, and Canada and may cause economic yield losses in oilseed rape production in Europe (Zeise and von Tiedemann, 2002; Debode et al., 2005; Depotter et al., 2016, 2019). *Verticillium longisporum* is an amphidiploid hybrid fungus forming three different lineages, A1/D1, A1/D2, and A1/D3, from four different haploid parental lines. Among them, A1/D1 is the most virulent lineage on oilseed rape (Tran et al., 2013; Depotter et al., 2016). After root infection, xylem invasion, and colonization of stems, *V. longisporum* forms melanized microsclerotia in the senescing tissues of the host plant, which remain viable for several years in the soil and therefore are a crucial survival mechanism (Mace et al., 2012; Zheng et al., 2019). However, regulation of dormancy is not only crucial for survival but also determines germination and successful host finding, and infection of roots. Since there is neither efficient chemical control nor effective cultivar resistance to control the disease, possible control strategies may be achieved by regulating the germination and dormancy of microsclerotia in the soil. However, knowledge about these regulating factors is surprisingly scarce.

In a single earlier study, Schreiber and Green (1963) showed that natural (unsterile) soil inhibited both conidia and microsclerotia of *Verticillium albo-atrum* when cultured in agar discs that had been placed in contact with soil for 24–36 h. The above mentioned study, for the first time, indicated the importance of natural soil to microsclerotia dormancy. However, since the “absence of soil” served as control in this study, the specific effect of soil microorganisms or other soil factors remained unclear. Soil is an important and complex habitat for numerous fungi, bacteria, protozoa, and other organisms (Dubey et al., 2019; Legrand et al., 2019). Several studies reported that natural soil can be suppressive to certain soil-borne pathogens, such as *Gaeumannomyces graminis* var. *tritici*, *Fusarium oxysporum,* and *Phytophthora* spp. (Kwak and Weller, 2013; Legrand et al., 2019; Ossowicki et al., 2020). One form of soil suppressiveness is soil fungistasis, which has been defined by Watson and Ford (1972) as a phenomenon inhibiting germination or growth of viable fungal propagules or fungal hyphae by conditions of the soil environment other than temperature and moisture, thus being caused by the soil microbiome rather than physicochemical factors in soil (Legrand et al., 2019). There have been primarily two kinds of soil fungistasis theories suggested, the nutrient deprivation hypothesis and the antibiosis hypothesis, and both of them have been supported by various experiments. The strongest evidence for the nutrient deprivation hypothesis is that the addition of nutrients (mainly sugars or amino acids) could partially or completely relieve the fungistasis effect (de Boer et al., 2003; Garbeva et al., 2011; Ossowicki et al., 2020). However, recent detection and identification of several bacterial volatile inhibitors of fungi also provided solid support for the antibiosis hypothesis (de Boer et al., 2019; Li et al., 2020). In addition, the impact of soil moisture on microsclerotia germination is usually regarded as an important factor affecting soil-borne plant pathogens (Cook and Papendick, 1972; Schuh et al., 1987).

A better understanding of the underlying mechanisms regulating dormancy and germination of microsclerotia in soil may provide us a novel strategy for preventing and controlling the diseases caused by Verticillium pathogens. One potential strategy can be to retain microsclerotia dormancy even in the presence of host plants, so that microsclerotia will not be able to germinate and attack the roots of their hosts. Another possibility is to artificially induce microsclerotia germination in the soil when host plants are absent during crop rotation cycles, reducing microsclerotia in soil by nutrient exhaustion due to failure to find a suitable target crop (Garbeva et al., 2011; Bonanomi et al., 2017). In either case, factors controlling microsclerotia dormancy or germination must be understood and identified.

Therefore, the overall aim of this study was to identify the factors in soil regulating dormancy and germination of *V. longisporum* microsclerotia in order to fill a crucial knowledge gap in the life cycle of this pathogen as well as to gain alternative ways to manage the disease caused by *V. longisporum*. More specifically, the present study aimed at (i) investigating the role of soil microorganisms, soil moisture, and nutrients in regulating microsclerotia dormancy and germination and (ii) identifying microbial compounds in soil with an inhibitory potential against *V. longisporum*.

## Materials and methods

### *In vitro* tests of soil fungistasis effect on microsclerotia

#### Production of microsclerotia

*Verticillium longisporum* isolate VL43 (lineage A1/D1) was used for microsclerotia production (Zeise and von Tiedemann, 2002). The isolate was grown on potato dextrose agar (PDA) at room temperature in the dark. An autoclavable spawn bag (PP75/BEU6/X32-57, SacO2, Belgium) containing a 14:1 quartz sand and rye flour mixture, and 9% of distilled water was autoclaved twice with a 24-h interval. One centimeter diameter mycelial agar plugs (about one agar plug per 225 *g* sand-flour mixture) from growing edges of fungal colonies were placed in the bag and incubated at room temperature in dark for approximately 3 weeks until dark-colored microsclerotia were formed on the surface of the sand-flour mixture. After drying the samples for 7 days at 25°C in a drying oven (Memmert GmbH + Co. KG, Schwabach, Germany), the sand-flour mixture with microsclerotia was passed through sieves to separate microsclerotia smaller than 100 μm and 100–200 μm in diameter. Microsclerotia were stored at 4°C under dark and dry conditions. Before use, microsclerotia were tested on lysogeny broth (LB) agar plates for bacterial contamination. If contamination was detected, microsclerotia were sterilized in an antibiotic solution containing 50 mg/l chlortetracycline, 50 mg/l chloramphenicol and 200 mg/l streptomycin for 2 h.

#### *In vitro* assays to analyze effects of nutrients on dormant microsclerotia

In order to examine whether nutrients are vital for inducing germination of microsclerotia, *ca.* 15 dormant microsclerotia (100–200 μm) were exposed to sterile water, PDB and glucose solutions in microscope slides with cavities (Thermo Fisher Scientific) at room temperature as well at under three different temperature levels in temperature cabinets (Rumed4000, Rubarth Apparate GmbH, Germany). Water and PDB were autoclaved at 121°C for 20 min and glucose solutions were sterilized by filtration (0.2 μm syringe filters, Sartorius stedim biotech, Germany). Germination of microsclerotia was scored every 2 h using an optical microscope (Leica Microsystems GmbH, Germany). The experiment was repeated twice with three replicates.

#### *In vitro* assays to analyze effects of soil moisture and soil sterility on dormant microsclerotia

Soil samples were randomly collected from the top layer (2–10 cm) at two different locations, an oilseed rape field in Weende, Göttingen, Germany (named as field soil) and a campus lawn at the University of Göttingen, Germany (named as grassland soil). Soil was sieved through a 2 mm sieve before use, and one half of fresh soil was sterilized twice at 121°C for 20 min with an overnight interval. The maximal water holding capacity (MWHC) of each soil was determined according to the following equation (Viji and Rajesh, 2012):


$$\begin{array}{l} Waterholdingcapacity=\frac{Totalwaterinthe\;wet\;soil}{Oven\;dry\;weightoftotalsoil}\\ \times 1\text{0}0 \end{array}$$

Based on this calculation, soil moisture was adjusted to 6%, 50%, and 90% MWHC by adding the required amount of distilled water. Twelve microsclerotia were spread on the surface of a cellulose acetate membrane (0.4 μm pore size, Sartorius Stedim Biotech GmbH, Germany), covered with another piece of membrane and buried into either sterile or unsterile soil. After 2 days of exposure, the membrane samples containing microsclerotia were carefully removed from the soil and stained with 0.25% Coomassie brilliant blue R250 in a methanol/acetic acid solution (Thermo Fisher Scientific). The germination rate was determined under a stereoscope (Leica Microsystems GmbH, Germany). The experiment was conducted twice with three replicates.

### *In vivo* tests of soil fungistasis effects on disease development

#### Inoculation experiment in the greenhouse

The effect of soil sterilization on infection of *B. napus* induced by microsclerotia was studied in a greenhouse experiment laid out in a completely randomized design. Seeds of the susceptible oilseed rape inbred line *B. napus* L. cv. Falcon (Norddeutsche Pflanzenzucht Hans-Georg Lembke KG, NPZ, Hohenlieth, Germany) were surface sterilized in 70% ethanol for 10 min, then treated with 1% sodium hypochlorite for 10 min, and washed three times with sterile distilled water. Seeds were placed on moist sterile quartz sand for 2 weeks to induce germination and then transferred to either sterile or unsterile field soil in 8 cm × 8 cm × 9 cm square-sided pots. For sterile conditions, the field soil was autoclaved twice at 121°C for 20 min with an overnight interval. Inoculation was done by adding 400 mg microsclerotia to 1 kg of soil. Plants were grown in a previously ozone-sterilized environmentally controlled chamber at 23/17°C (day/night), 16/8 h light, and a relative humidity of 70%. The plants were watered with autoclaved tap water once a day and fertilized with HAKAPHOS^®^ “Blau” 4 weeks after transplanting. The experiment was conducted twice with 20 replicates.

#### Visual assessment of disease development

Plant height was recorded and disease symptoms were scored every week using a scoring system modified from Zeise (1992). The area under the disease progress curve (AUDPC) was calculated according to the formula below, with the parameters *y_i_* (disease severity score), *t_i_* (number of days post inoculation) and *n* (total number of observations; Eynck et al., 2007):


$$AUDPC=\overset{n}{\underset{i=1}{\sum }}\left(\frac{y_{i}+y_{i+1}\;}{2}\right)\times \left(t_{i+1}+t_{i}\right)$$

The net value of AUDPC (*AUDPC_net_*) was determined as difference between AUDPC of inoculated plants (*AUDPC_inoc_*) and of control plants (*AUDPC_cont_*):


$$AUDPC_{net}=AUDPC_{inoc}-AUDPC_{cont}$$

#### Quantification of fungal biomass with real-time PCR

Plant hypocotyls from all treatments were harvested 4 and 10 weeks after treatment (WAT) and carefully washed. Samples were lyophilized and ground with a swing mill (MM 400, Retsch GmbH, Haan, Germany) for 1 min at 30 Hz. Total DNA was extracted from plant samples using the cetrimonium bromide (CTAB) method (Brandfass and Karlovsky, 2008). The quality of the DNA was examined with 1% agarose gel electrophoresis and NanoDrop (Epoch, BioTek, United States). Subsequently, the DNA was stored at −20°C until use. Real-time PCR analysis for quantification of fungal DNA was carried out using the primer pair OLG 70 (5′CAGCGAAACGCGATATGTAG 3′)/OLG 71 (5′GGCTTGTAGGGGGTTTAGA 3′), which targets the ribosomal internal transcribed spacer (ITS) region of *V. longisporum* (Eynck et al., 2007). A primer pair GAPDH_F (5′CGCTTCCTTCAACATCATTCCCA 3′)/GAPDH_R (5′TCAGATTCCTCCTTGATAGCCTT 3′) was applied to amplify the *GADPH* gene of oilseed rape, which was used as internal standard to normalize differences between DNA samples. The PCR reaction was carried out in the iCycler System (BioRad, Hercules, CA, United States). The PCR program included an initial 3 min denaturation step, followed by 40 cycles of 94°C for 1 min, 60°C for 1 min, and 72°C for 30 s and was performed with 10 μl reaction mixtures consisting of 5 μl Master Mix (qPCRBIO SyGreen Mix Lo-Rox, Nippon Genetics Europe GmbH), 0.3 μM of each primer, 1 μl of template DNA, and 3.4 μl ddH_2_O. The real-time PCR results were analyzed using the CFX Manager Software (BioRad, Hercules, CA, United States).

For the quantification of pathogen or plant DNA, a base-10 semi-logarithmic standard curve was generated by plotting the C_t_ values versus the dilution factor (10-fold serial dilutions of DNA template) of pure *V. longisporum* or *B. napus* L. cv. Falcon DNA samples. The standard curve was run in parallel reactions to determine the amount of pathogen DNA in oilseed rape samples, and the results were demonstrated as pg. of fungal DNA in 1 ng of total DNA (plant DNA + fungal DNA) extracted from each sample.

### Bioassays to evaluate the effect of soil bacteria on microsclerotia

#### Bacterial strains

Eighteen strains of soil bacteria were isolated from the rhizosphere of oilseed rape plants and field bulk soil (Weende, Göttingen, Germany). One gram of each soil sample, collected either from soil adhering to roots of field-grown plants or from the top layer of bulk field soil (5–10 cm), was dispersed in 10 ml of sterilized water and a serial dilution of it was prepared. An aliquot of 100 μl of soil suspension was then inoculated on LB growth medium and according to their colony form, bacteria were roughly categorized and labelled, and each single colony culture (5 × 10^8^ CFU/mL) was stored at −80°C in 25% glycerol for further use. In addition, wild type strains of *Escherichia coli* and *Acidovorax valerianellae* were provided by Dr. Athanassios Mavridis, University of Göttingen, and *Bacillus subtilis* wild type strain JH642 was obtained from Prof. Dr. Dieter Jahn, Institute of Microbiology, Technical University of Braunschweig.

#### Bioassay with bacteria suspension and microsclerotia

For a treatment with living bacteria, 100 μl bacteria suspension stored at −80°C was incubated in 6 ml liquid LB medium overnight. For a treatment with dead bacteria and bacterial culture filtrate, the above mentioned bacterial suspensions were autoclaved twice (121°C for 20 min) or filtered through 0.2 μm syringe filters (Sartorius stedim biotech, Germany), respectively. The concentration of bacterial suspension was determined according to the correlation between OD600 extinction (optical density of bacteria sample measured at the wavelength of 600 nm) and number of colony-forming units (CFU) per mL LB medium. Two soil bacteria (named as soil bacteria1 and soil bacteria2), as well as strains of *E. coli* and *B. subtilis* were selected to evaluate the effect of dead bacteria and bacterial culture filtrate on microsclerotia germination. This bioassay was conducted with the same method as the assessment of temperature and nutrient effects on dormant microsclerotia and was repeated twice with 4 replicates.

#### Bioassay with bacterial volatiles and microsclerotia

The effect of bacterial volatiles on the germination of microsclerotia was studied using two-compartment Petri dishes as described by Cordero et al. (2014), with minor modifications. Two-compartment Petri dishes assay is a main way to study inhibiting activities of bacterial volatiles since this method allows to determine effects of bacteria on other organisms without direct physical contact (Tyc et al., 2015; Li et al., 2020). Bacteria used in this experiment were soil bacteria1, soil bacteria2, *E. coli*, and *B. subtilis*. One compartment loaded with LB agar was inoculated with 100 μl bacterial stock suspension (5 × 10^8^ CFU/mL) or their 100-, 10,000-, or 1,000,000-fold dilutions. Plates were sealed and incubated at 25°C for 3 days and then microsclerotia were spread in the opposite compartment loaded with water agar (WA). The LB medium was amended with 50 mg/l cycloheximide (Sigma-Aldrich, St. Louis, MO, USA) and 50 mg/l thiabendazole (Sigma-Aldrich, location) to inhibit fungal growth and the WA medium was added with 200 mg/l streptomycin (Sigma-Aldrich, location) to suppress bacterial growth. The Petri dishes were sealed again and incubated further at 25°C. The diameter of fungal colonies was measured 7 days after treatment (DAT). The experiment was repeated twice with three replicates.

### Bioassays to evaluate the effect of pure individual VOC on microsclerotia

#### Sampling of VOCs and analysis by GC–MS

For the sampling of bacterial VOCs, 100 μl bacterial suspensions were inoculated in 40 ml of LB medium in a 100 ml extraction vial, especially designed for volatile extraction with one inlet and one outlet. After incubation at 25°C (soil bacteria 1 and soil bacteria 2) and at 37°C (*B. subtilis* and *E. coli*) for 3 days, samples were used for extraction. LB medium without bacteria was used as a control. Volatile collection traps containing 30 mg Porapak-Q adsorbent (Volatile Collection Trap LLC, FL, United States) were attached to the outlet of the vial as described previously (Rostás and Eggert, 2008). Filtered (activated charcoal filter, 400 ccs, Alltech, Deerfield, IL, United States) and humidified air was pushed into each vessel by the inlet of the vial at a rate of 0.5 l per min originating from a central in-house compressor. With a vacuum pump (N816.3KN.18, Laboport^®^, Germany), the same amount of air (0.5 l/min) was pulled through the trapping filter attached to the outlet of the vial and volatiles were collected for 3 h.

After collection, the trapped volatiles were eluted with 150 μl dichloromethane (DCM) in 1 ml glass vials and subsequently stored at −80°C for further analysis. Before gas chromatography–mass spectrometry (GC–MS) analysis, 200 ng of tetralin (1, 2, 3, 4 tetrahydronaphthalene, Sigma-Aldrich, Taufkirchen, Germany) was added to each sample as an internal standard. Later, 30 μl of each sample was transferred to another vial for immediate use or stored at −80°C for further study. The qualitative and quantitative composition of VOCs of each sample was analyzed using an Agilent Technologies GC (GC 7890B, MS 5977B). Samples of 2 μl volume were injected with an automated injection system in pulsed splitless mode. The oven temperature was held at 40°C for 3 min and then increased gradually to a final temperature of 220°C, which was held for 10 min. Helium (1.5 ml min/mL) was used as the carrier gas. The software MSD ChemStation with the NIST17 and Wiley11 mass spectral libraries was used to identify compounds by their mass spectra and retention indices. The experiment was conducted twice with three replicates and compounds detected in at least 3 replicates were regarded as identified compounds. Compound quantification was achieved by comparing their areas to the area of the internal standard.

#### Bioassay with individual VOCs and microsclerotia

The antagonistic effects of pure VOCs against microsclerotia were assessed in two-compartment Petri dishes using a similar experimental design as described above. According to results from the GC–MS analysis, the most abundant 9 VOCs, 2,5-dimethylpyrazine, acetoin, phenylethyl alcohol, 3-methyl-1-butanol, 2-methyl-1-butanol, 3-methylbutanoic acid, 2-methylbutanoic acid, hexanoic aicd, 2-methylpropionic acid, were selected for this assay. In addition, 2,3-butanediol was selected as this bacterial volatile is considered as important bioactive compound involved in plant resistance against several plant pathogens (D’alessandro et al., 2014; Kai, 2020). All pure chemicals were purchased from Sigma-Aldrich Company. The pure compounds were dissolved in dimethyl sulfoxide (DMSO). Then, 100 μl of each compound at different concentrations (5 M, 1 M, 500 mM, and 250 mM) was added to filter discs (MN 827 ATD, Macherey-Nagel, Germany) placed in one compartment and microsclerotia were plated on WA in the opposite compartment. Pure DMSO and clean filter discs without any chemicals served as control. Petri dishes were sealed and incubated at 25°C. The diameter of fungal colonies was measured 7 DAT. The experiment was repeated twice with three replicates.

### Data analysis

Data were analyzed either with one-way ANOVA or Kruskal–Wallis tests at the significance level of 0.05 (*p* < 0.05). Tukey’s HSD (honestly significant difference) test and Pairwise Wilcox test (value of p correction with the Benjamini–Hochberg procedure) were applied to perform pairwise comparisons between groups. Statistical tests were carried out with the software R version 4.0.4 (R Development Core Team) and RStudio Desktop version 1.0.153 (RStudio Inc. Boston, United States).

## Results

### *In vitro* effects of nutrients, soil microbes, and moisture on microsclerotia germination

**Figure 1:**
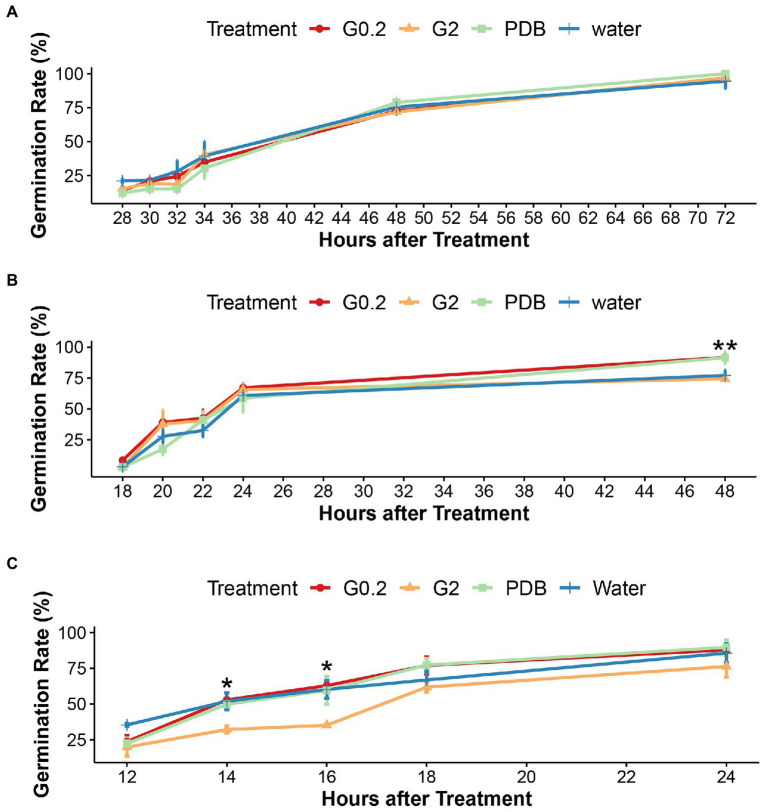
Effects of water and nutrients on germination of microsclerotia **(A)** at 10°C, **(B)** at 15°C, and **(C)** at 20°C, under sterile conditions *in vitro.* G0.2, G2, and PDB indicates 0.2%, 2% glucose solution, and Potato Dextrose Broth, respectively. Error bars indicate standard error of the mean (*n* = 3), ^**^indicates a statistically significant difference at *p* < 0.01, ^*^indicates a statistically significant difference at *p* < 0.05 (ANOVA test).

The cultivation of microsclerotia in sterile double distilled water, PDB, and glucose solutions (0.2%, 2%) showed that microsclerotia could well germinate in sterile distilled water. No significant differences in germination rates between different treatments were detected at room temperature and at 10°C (Supplementary Figure S1 and Figure 1A). At 15°C and 48 h after treatment (HAT), germination rates in water and 2% glucose were significantly lower than in the other two media (Figure 1B). At 20°C, the germination rate of microsclerotia in 2% glucose solution was significantly lower than in the other treatments at 14 and 16 HAT (Figure 1C). In the given temperature range from 10 to 20°C, the results from 12, 18, and 28 HAT indicated a positive correlation between temperature and start of germination (Figure 1).

**Figure 2:**
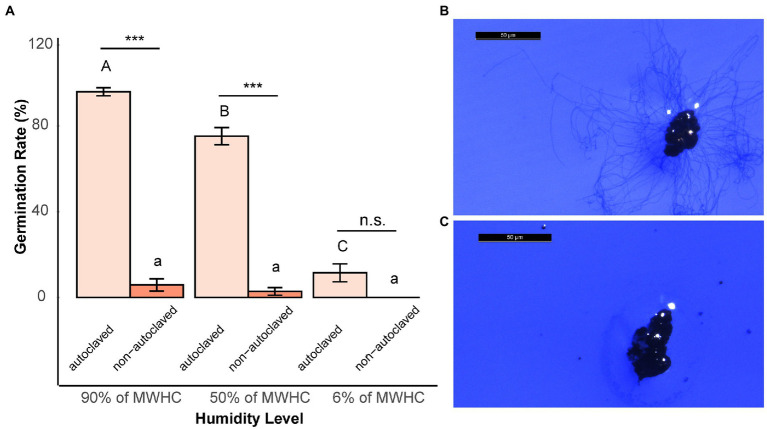
**(A)** Germination rate of microsclerotia after 2 days of incubation in sterile and unsterile field soil at different soil humidity levels. MWHC indicates maximal water holding capacity. ^***^indicates a statistically significant difference at *p* < 0.001 between different soil treatments at the same humidity level (ANOVA test). Different letters indicate significant differences (*p* < 0.05) between different humidity levels of same soil treatment. Error bars indicate standard error of the mean (*n* = 3; Tukey’s test). The photos show germinated **(B)** and dormant **(C)** microsclerotia under stereoscopic microscope (scale bar: 50 μm).

Fungistatic effects of soil bacteria on microsclerotia were investigated by cultivating microsclerotia in presence of autoclaved and non-autoclaved soil at different humidity levels. The results of both field soil and grassland soil showed the same effects (Figure 2 and Supplementary Figure S2). Under higher soil humidity (90% and 50% of MWHC), the germination rate in autoclaved soils was significantly higher than in non-autoclaved soils. However, in general, germination was significantly reduced at the low humidity level of 6% of MWHC where it did not differ between autoclaved and non-autoclaved treatments. The germination rate of microsclerotia in sterile soil declined along with the decrease in humidity levels, indicating the importance of water in the germination of microsclerotia (Figure 2 and Supplementary Figure S2).

### Effect of soil sterility on disease severity and pathogen growth *in vivo*

**Figure 3:**
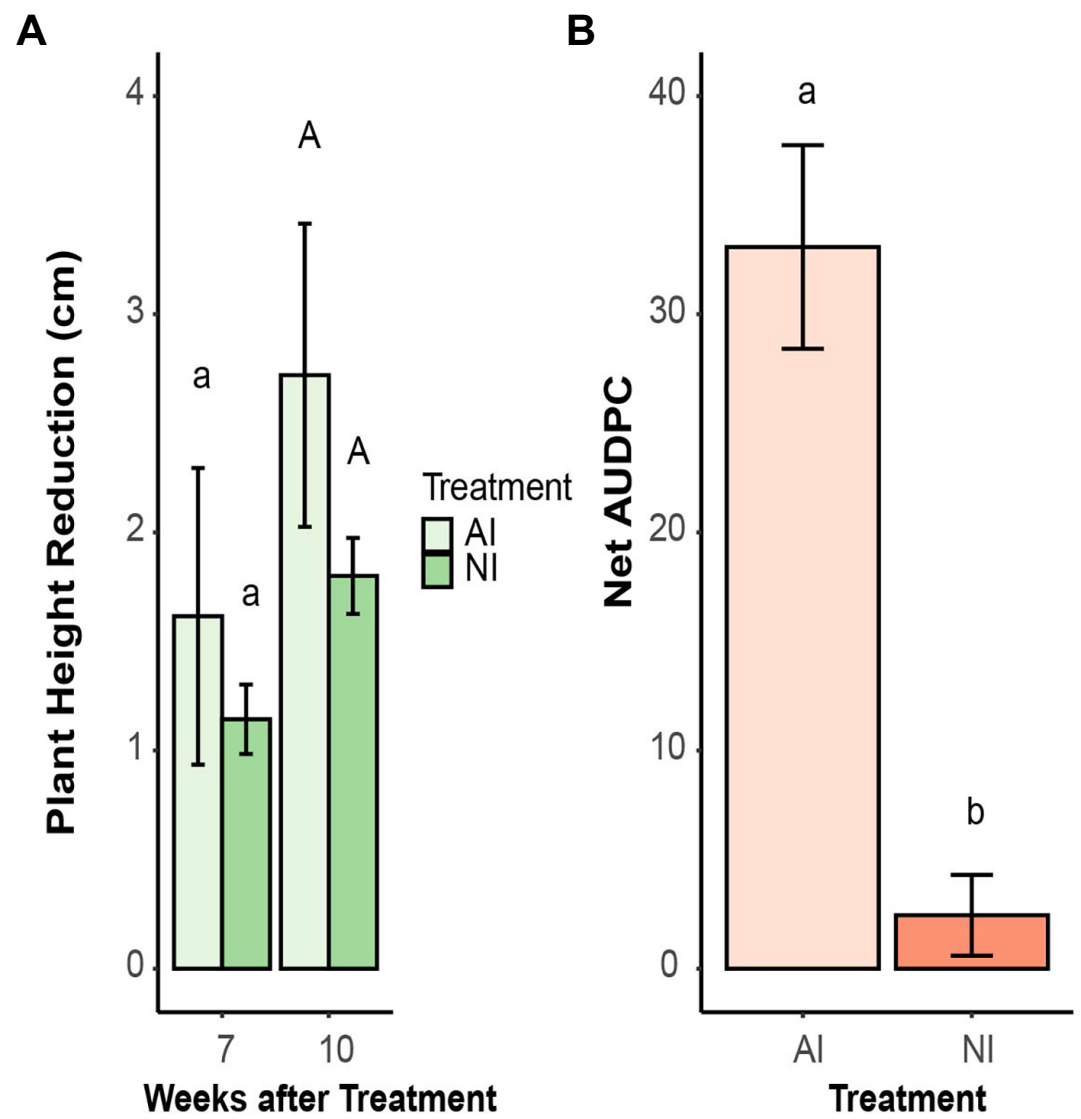
**(A)** Plant height reduction of oilseed rape plants inoculated with microsclerotia of *V. longisporum* compared to their non-inoculated control plants. Plants were grown in autoclaved (AI) and non-autoclaved (NI) soil. Different lowercase and uppercase letters indicate significant differences (*p* < 0.05) between different soil treatments after 7 and 10 weeks, respectively. Error bars indicate standard error of the mean (*n* = 20). **(B)** Net area under the disease progress curve (AUDPC) of oilseed rape plants inoculated with microsclerotia of *V. longisporum* and grown AI and NI soil. Different letters indicate significant differences (*p* < 0.05) between different soil treatments. Error bars indicate standard error of the mean (*n* = 20; Kruskal–Wallis test).

There was no difference in growth reduction due to infection between autoclaved and non-autoclaved soils (Figure 3A). However, the disease scoring revealed that inoculated plants grown on autoclaved soil had significantly higher AUDPC values than on non-autoclaved soil (Figure 3B).

**Figure 4:**
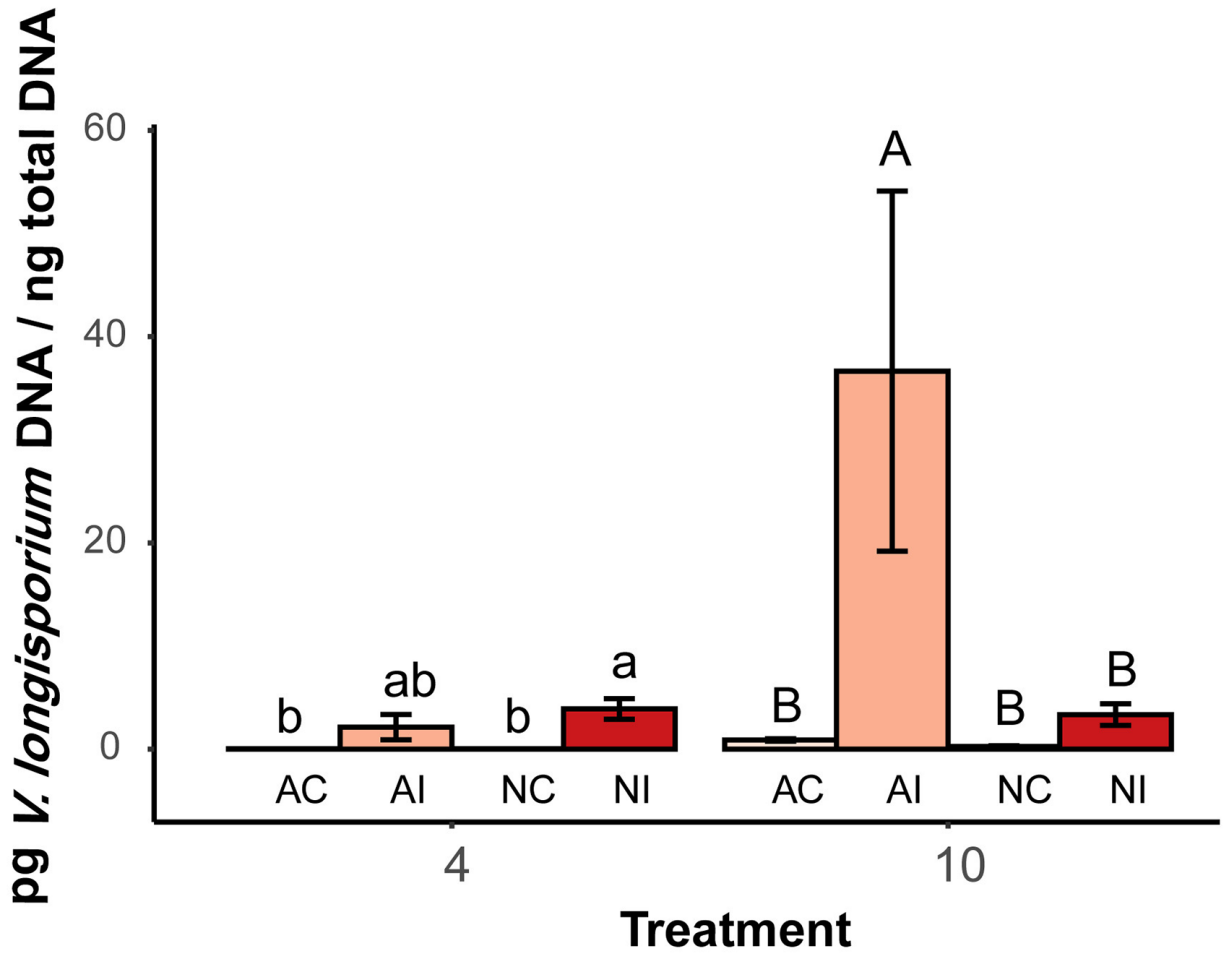
Quantities of *V. longisporum* DNA in hypocotyl of oilseed rape plants at two time points (4 and 10 weeks after inoculation) with microsclerotia of *V. longisporum*. AC indicates control plants grown AI soil, AI indicates inoculated plants grown AI soil, NC indicates control plants grown in NI soil and NI indicates inoculated plants grown in NI soil. Different lowercase and uppercase letters indicate significant differences (*p* < 0.05) between different treatments after 4 and 10 weeks, respectively. Error bars indicate standard error of the mean (*n* = 3; Tukey’s test).

The growth of *V. longisporum* in the hypocotyl of *B. napus* was quantified by real-time qPCR. Significantly higher amounts of *V. longisporum* DNA were detected in plants grown in autoclaved soil compared to plants grown in non-autoclaved soil. At the first time point (4 WAT), there was no difference between autoclaved and non-autoclaved treatment. However, 10 WAT, the amount of pathogen DNA in plants grown in autoclaved soil, was significantly higher (34.1 pg. per ng total DNA) than in plants grown in non-autoclaved soil [2.67 pg. VL per ng total DNA (Figure 4)].

### Effects of bacterial suspensions and VOCs produced by bacteria

**Figure 5:**
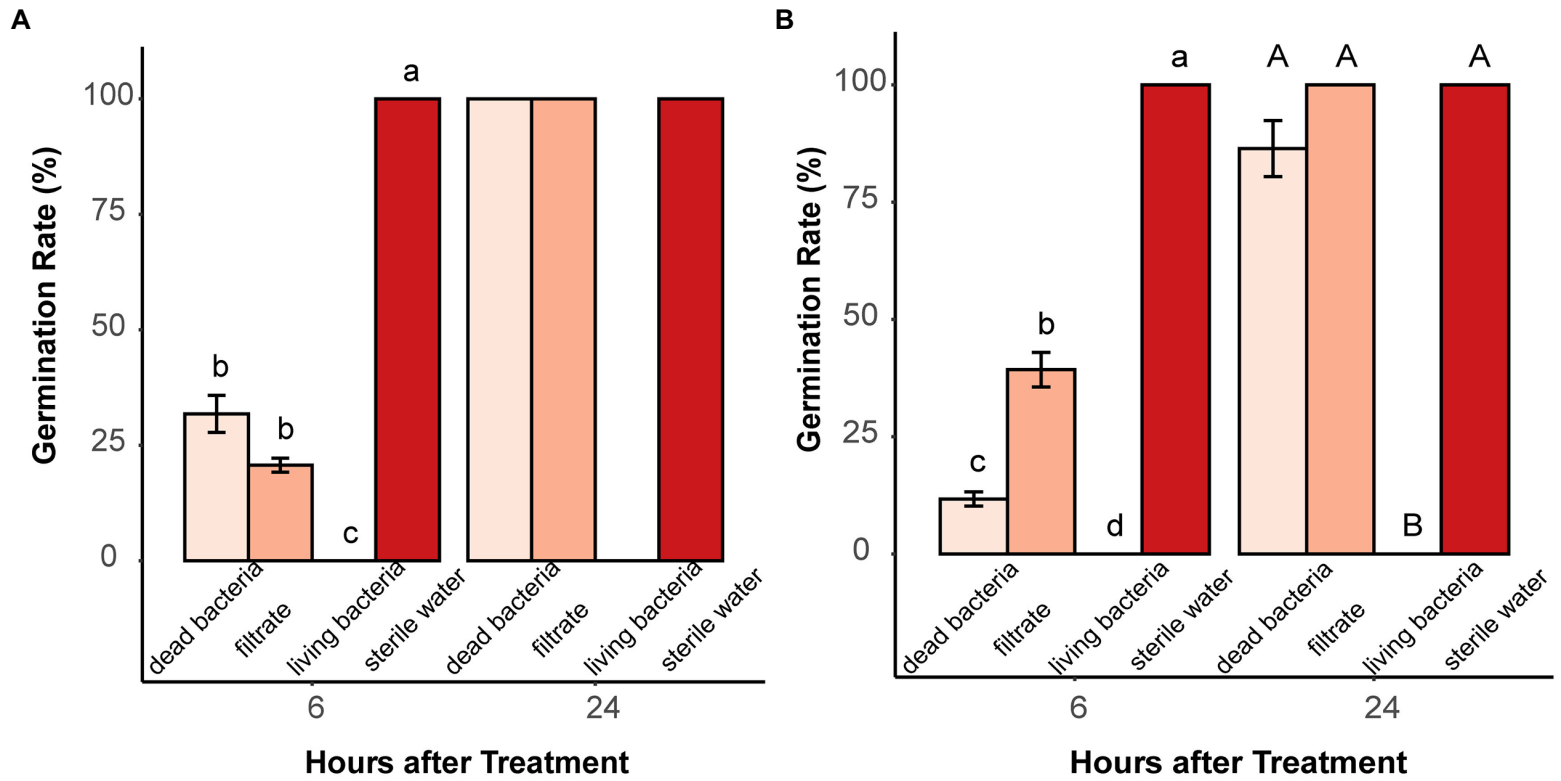
Effect of dead bacteria, bacterial culture filtrate and living bacteria on microsclerotia germination. **(A)** Soil bacteria 1 and **(B)** *Bacillus subtilis.* Different lowercase and uppercase letters indicate significant differences (*p* < 0.05) between treatments 6 and 24 h after treatment, respectively. Error bars indicate standard error of the mean (*n* = 4; Pairwise Wilcox test).

In total, 21 bacterial isolates (18 soil bacteria, *E. coli*, *B. subtilis*, and *A. valerianellae*) were tested for their inhibitory effects on the germination of microsclerotia. All tested bacteria suppressed the germination of microsclerotia at a concentration of 5 × 10^8^ CFU/ml. For *E. coli*, 16- and 100-fold dilutions of bacterial suspension almost lost their inhibitory effect at 12 HAT (Supplementary Figure S3). Furthermore, incubation of microsclerotia with dead bacterial suspension and culture filtrate solution of *B. subtilis, E. coli* and two randomly selected soil bacteria (soil bacteria1 and soil bacteria 2) only partially suppressed the germination of microsclerotia at 6 HAT and had no inhibitory effect at 24 HAT (Figure 5 and Supplementary Figure S4).

**Figure 6:**
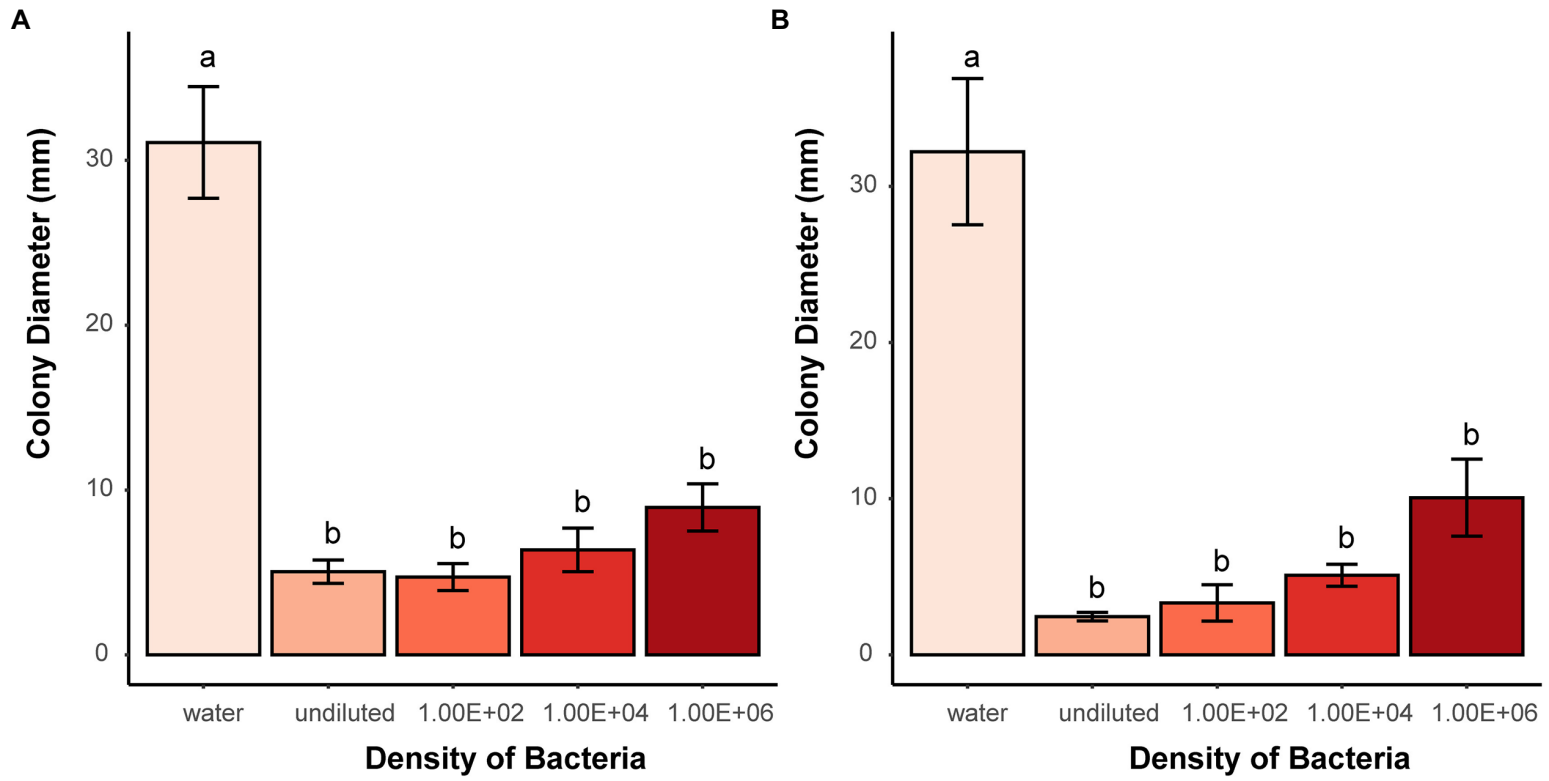
Effects of bacterial volatiles on the colony growth originating from microsclerotia after 7 days of incubation. **(A)** Soil bacteria 1 and **(B)** *B. subtilis.* Undiluted means stock suspension of bacteria without dilution. Numbers indicate 100, 10,000, and 1,000,000 times dilution of stock suspension. Different letters indicate significant differences (*p* < 0.05) between treatments. Error bars indicate standard error of the mean (*n* = 3; Tukey’s test).

Volatiles produced by living bacteria and tested in two-compartment Petri dishes demonstrated that they can effectively prevent microsclerotia from germinating. Compared to the water control, the average diameter of the colonies formed around the microsclerotia was significantly reduced in all dilutions of *B. subtilis*, soil bacteria1, and *E. coli* or in higher concentrations of soil bacteria 2 (Figure 6 and Supplementary Figure S5).

### Identification and antifungal activity of VOCs

**Figure 7:**
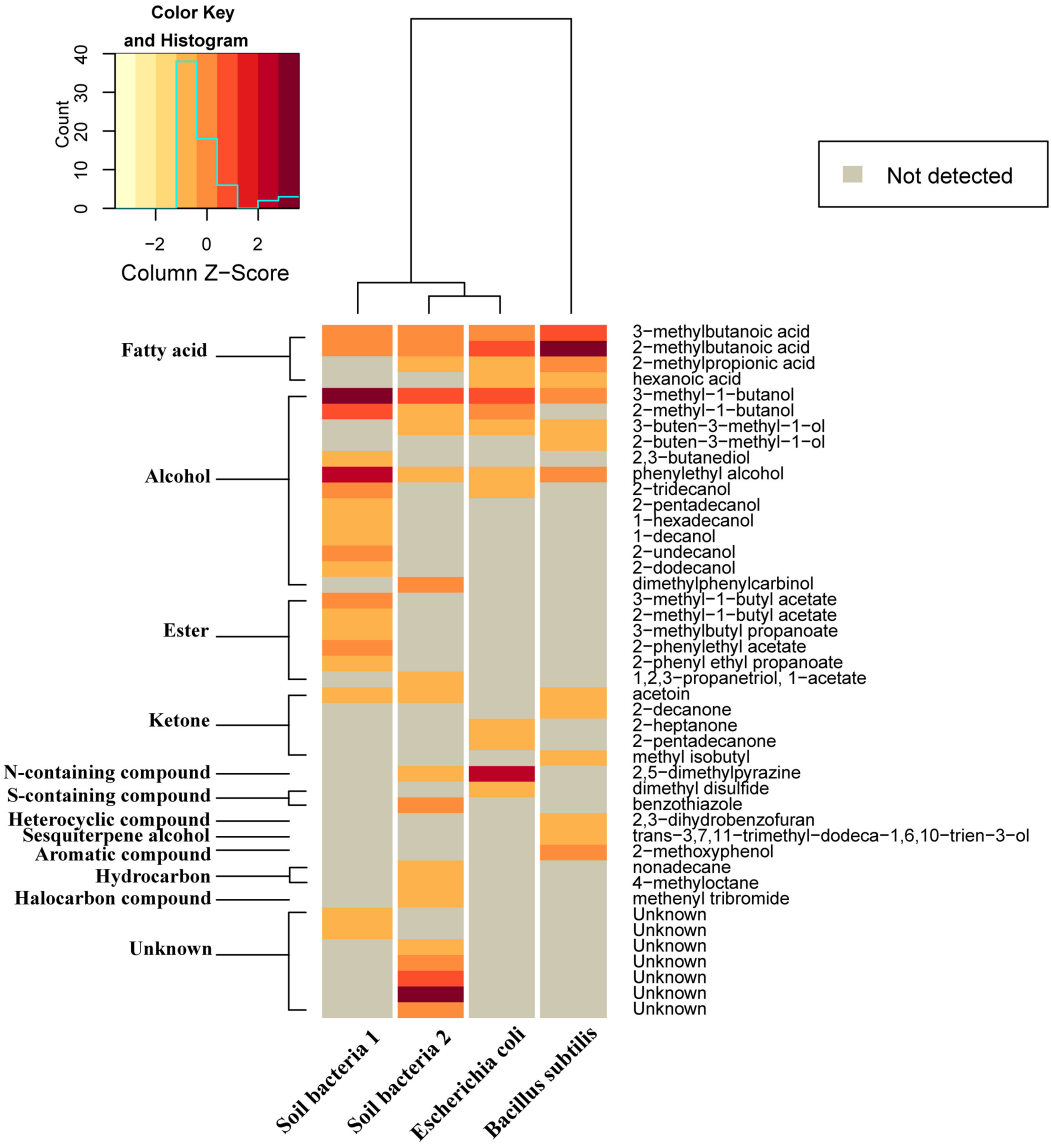
Relative amounts of volatile compounds emitted by four different bacteria displayed as a heat map generated based on Euclidean distance using Ward algorithm and showing average amounts of different compounds (ng/h/CFU). Gray color indicates quantities below detection threshold.

In total, 45 VOCs of 11 different chemical groups were identified in four different bacterial isolates. The most abundant VOCs were alcohols, followed by ketones, esters, and fatty acids. The pattern of VOCs differed quite substantially among bacteria. However, four VOCs were detected in all four bacterial isolates. These include two fatty acids (3-methylbutanoic acid and 2-methylbutanoic acid) and two alcohols (3-methyl-1-butanol and phenylethyl alcohol). In *E. coli*, the three most abundant volatile compounds were 2,5-dimethylpyrazine, 2-methylbutanoic acid, 3-methyl-1-butanol and in *B. subtilis*, the three prevalent compounds were 2-methylbutanoic acid, 3-methylbutanoic acid and phenol, 2-methoxyphenol. In soil bacteria1, 3-methyl-1-butanol, phenylethyl alcohol and 2-methyl-1-butanol were most abundant while in soil bacteria 2, two unknown compounds and 3-methyl-1-butanol were most frequently detected (Figure 7).

**Figure 8:**
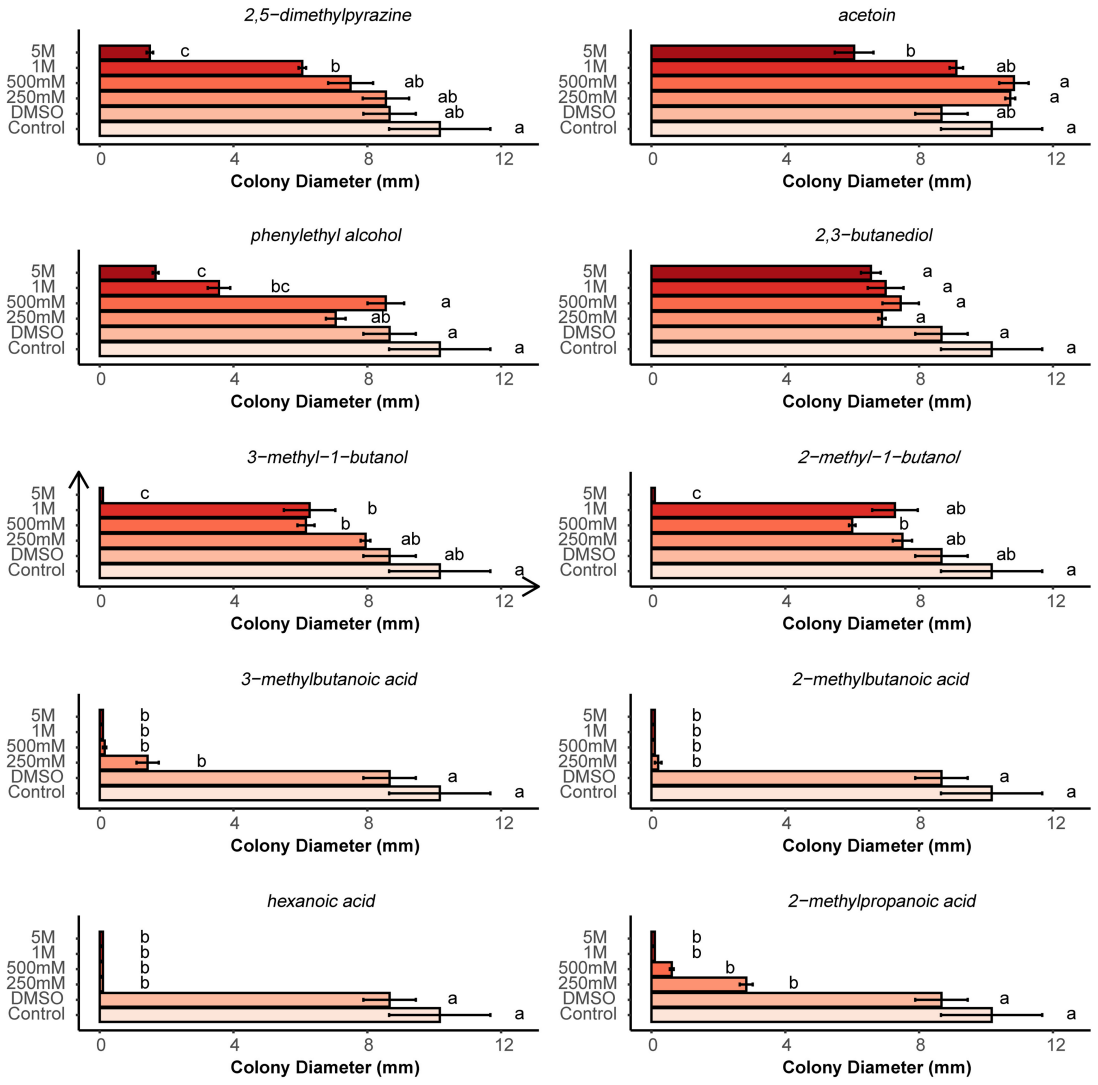
Effects of different concentrations of pure organic volatiles dissolved in DMSO on the colony growth of germinating microsclerotia. Different letters indicate significant differences (*p* < 0.05) between treatments. Error bars indicate standard error of the mean (*n* = 3; Tukey’s test).

According to abundance and frequency of occurrence, 10 VOCs were selected to determine their single antagonistic effect on microsclerotia using two-compartment Petri dishes. Compared with the control and pure DMSO, the growth of microsclerotia was completely or strongly inhibited at all tested concentrations of four fatty acid compounds (3-methylbutanoic acid, 2-methylbutanoic acid, hexanoic acid, and 2-methylpropionic acid). Acetoin, 2-methyl-1-butanol, 2,5-dimethylpyrazine and phenylethyl alcohol significantly suppressed the growth of microsclerotia only at high concentrations. 3-Methyl-1-butanol was able to significantly suppress the growth of microsclerotia at 500 mM and higher concentrations. 2,3-Butanidiol showed no antagonistic effect against microsclerotia (Figure 8).

## Discussion

Staying viable in the soil for several years in form of microsclerotia and germinating once the host plants are present is a crucial survival mechanism of Verticillium pathogens (Mace et al., 2012). Therefore, the factors regulating dormancy and germination of microsclerotia are key for successful completion of their life cycle. Soil fungistasis has been considered a main mechanism for keeping soil-borne pathogens in a dormant state (Watson and Ford, 1972). Our data from *in vitro* as well as *in vivo* assays provide evidence, that soil fungistasis also inhibits microsclerotia of *V. longisporum.* In the present study, dormant microsclerotia were cultured with a range of sterile nutrient solutions as well as autoclaved distilled water to test whether carbon-rich nutrients may break dormancy faster than water. The results showed that there was no difference between the stimulating effect of nutrients and water at different temperatures (Figure 1 and Supplementary Figure S1). Although at lower temperature the germination time point of microsclerotia was postponed, the strong stimulatory effect of water on microsclerotia germination was consistent throughout three different temperatures (Figure 1). This indicates that the availability of external carbon sources is not a limiting factor of microsclerotia germination and implies a possible role of inhibitory soil microbes.

The strong inhibitory effect of soil microbes on microsclerotia was confirmed by culturing microsclerotia in both autoclaved and non-autoclaved soil. At humidity levels of 90% and 50% of MWHC, the germination of microsclerotia was strongly inhibited in unsterile natural soil, while the majority of microsclerotia germinated in autoclaved soil (Figure 2 and Supplementary Figure S2). Notably, germination of microsclerotia was significantly reduced also in autoclaved soil when soil moisture decreased. This indicates that apart from fungistasis, soil moisture plays a vital role in germination/dormancy of microsclerotia.

The soil fungistasis effect on microsclerotia germination was further confirmed *in vivo* with oilseed rape plants inoculated with *V. longisporum*. Both, visual disease scoring and fungal colonization of stems quantified by qPCR analysis showed that plants grown in autoclaved soil were significantly stronger affected by the pathogen than plants grown in non-autoclaved soil (Figures 3, 4). Stunting is a significant disease symptom of oilseed rape infected with *V. longisporum* under greenhouse conditions (Zeise and von Tiedemann, 2002). In the present study, inoculated plants grown in autoclaved and non-autoclaved soil showed reduced plant growth when compared with their control plants (Supplementary Figure S6). However, inoculated plants from autoclaved soil did not show a significantly larger reduction in height than those from the non-autoclaved treatment, as one would expect (Figure 3A). Since the control plants in sterile soil were significantly higher than control plants in non-sterile soil, one possible explanation could be that soil sterilization had a growth-enhancing effect on oilseed rape and this has compensated for the impairment caused by disease (Supplementary Figure S6).

The experiments *in vivo* and *in vitro* imply that removal of viable soil microorganisms induces germination of microsclerotia and aggravates disease severity. Rybakova et al. (2017) reported that seeds of different oilseed rape cultivars have distinct bacterial communities, and those cultivars with a higher indigenous bacterial diversity tend to be more resistant to potential pathogens.

There exist two theories to explain the fungistatic mechanisms underlying inhibition of germination and proliferation of soil-borne pathogens. One is the nutrient deprivation hypothesis, saying that limited carbon availability in the soil is the main factor preventing germination of resting structures of soil-borne fungi (de Boer et al., 2003; Garbeva et al., 2011). However, the *in vitro* nutrient test conducted in the present study shows that the energy stored within microsclerotia is sufficient for their germination, and external nutrients had no effect on dormant microsclerotia (Figure 1 and Supplementary Figure S1). The antibiosis hypothesis, on the other hand, claims that germination and growth of soil-borne pathogens are governed by antifungal compounds originating from soil microbes (de Boer et al., 2003; Garbeva et al., 2011; Ossowicki et al., 2020).

In our study, *E. coli*, *B. subtilis*, *A. valerianellae*, and 18 other bacteria isolated from bulk and rhizosphere soil were tested for inhibitory effects on microsclerotia. Interestingly, all bacteria tested had a complete suppressive effect on germination of microsclerotia when their density was at least 5 × 10^8^ CFU/ml. At lower densities, the inhibitory effect on microsclerotia diminished and microsclerotia started to germinate (Supplementary Figure S3). Bacterial diversity in soil is estimated to comprise 10^4^ bacterial species and bacterial density is around 10^9^ bacteria per g soil in nature (Poole, 2017), which is sufficient to consistently suppress germination of microsclerotia as demonstrated in the present study. Microsclerotia were also exposed to dead bacterial suspensions as well as to bacterial cell-free culture filtrates. In presence of dead bacteria and bacterial filtrates, the germination speed was transiently slowed down, but after 24 h, the germination rate fully recovered (Figure 5 and Supplementary Figure S4). This may either indicate that the inhibiting substances from bacteria need to be constantly produced by living bacteria, or that they have a low solubility in water and poor heat stability. The earlier implies that the germination-inhibiting substances need to be emitted as volatiles from living bacteria.

Indeed, this assumption was confirmed in the present study using two-compartment Petri dishes. In the volatile receiving compartment, the growth of fungal colonies from microsclerotia was strongly reduced when sufficient amounts of bacteria were cultured in the neighboring compartment. Remarkably, the diameter of fungal colonies increased as the bacterial density was reduced, which was consistent with our previous results on reduced germination of microsclerotia exposed to viable bacterial suspensions (Figure 6 and Supplementary Figures S3, S5).

In recent years, several studies have revealed the inhibitory effect of bacterial volatiles on different fungal species. Zou et al. (2007) found that 328 bacterial isolates from 5 bacterial families produce antifungal volatile organic compounds (VOCs) that inhibit the germination of conidia and mycelial growth of *Paecilomyces lilacinus* and *Pochonia chlamydosporia*. Moreover, they found out that the soil with a stronger fungistatic effect contained a higher proportion of these fungistatic bacteria. Ebadzadsahrai et al. (2020) detected that 21 of 68 tested bacterial isolates displayed antifungal VOC activity against several fungal and oomycete plant pathogens. The VOC activity from different strains of *Bacillus* spp. towards different fungi has been also widely studied in recent years. Volatile compounds emitted from *Bacillus spp.* with antifungal effects are derived from various different chemical groups, including hydrocarbons, ketones, alcohols, aldehydes, esters, ethers, acids, aromatics, and other sulfur or nitrogen containing compounds (Kai, 2020; Zhang et al., 2020).

In order to identify the active volatile compounds suppressing germination of microsclerotia, they were collected from bacterial *in vitro* cultures and analyzed by GC–MS. After we found that all tested 21 bacterial isolates showed similar germination-inhibitory effects on microsclerotia, the VOCs commonly produced by four bacteria, *B. subtilis*, *E. coli*, and two randomly selected soil bacteria, were identified. Overall, four compounds, 2-methylbutanoic acid, 3-methylbutanoic acid, 3-methyl-1-butanol, and phenylethyl alcohol, were detected in the above mentioned four bacteria (Figure 7).

Ten VOC compounds with high common abundance were selected and purchased, and pure chemicals were tested for antifungal effects against microsclerotia. Interestingly, fatty acid VOCs had the strongest inhibitory effect on germination of microsclerotia (Figure 8). In total, four fatty acids were detected in our study and all were found to be emitted by bacteria in higher amounts (Figure 7). 2-Methylbutanoic acid and 3-methylbutanoic are detected in all four bacteria, 2-methylpropanoic acid was found in three bacteria isolates except for soil bacteria1, and hexanoic acid was identified in *B. subtilis* and *E. coli* (Figure 7).

Fatty acids are common natural substances that exhibit various specific functions related to energy storage and different cellular processes (Pohl et al., 2011; Gołębiowski et al., 2014). Several fatty acids have been reported to have antibacterial and antifungal activity. Because of their significant advantages over conventional antimicrobial drugs (e.g., low environmental impact, synergy with other antimicrobial compounds, high antimicrobial efficacy at lower pH etc.), these antimicrobial fatty acids have a great potential for application in a variety of industries, including agriculture (Desbois, 2012). 2-Methylbutanoic acid and 3-methylbutanoic are reported to inhibit the growth of the common bean pathogen *Colletotrichum lindemuthianum* (Martins et al., 2019). Carballeira (2008) reported that 2-hexadecynoic acid inhibited the growth of *Mycobacterium tuberculosis*. Linolenic and linoleic acids exhibited activity against the plant pathogens *Rhizoctonia solani*, *Pythium ultimum, Pyrenophora avenae*, and *Crinipellis perniciosa* (Walters et al., 2004). Lauric acid exerted strong bioactivity against *Fusarium* spp. (Altieri et al., 2009). Direct interaction with fungal cell membranes as well as interrupting several fungal cellular activities through inhibition of fungal N-myristoyltransferase, fatty acid metabolism or topoisomerase I was suggested as possible modes of action of these bioactive fatty acids (Pohl et al., 2011). However, the exact mechanism of antifungal effects of fatty acids is still unclear.

Six other compounds apart from fatty acids (2,5-dimethylpyrazine, acetoin, phenylethyl alcohol, 2,3-butanediol, 3-methyl-1-butanol, and 2-methyl-1-butanol) showed less or no antagonistic effects toward microsclerotia of *V. longisporum* (Figure 8). However, phenylethyl alcohol was reported to strongly inhibit the mycelial growth and sporulation of *Peronophythora litchii* (Xing et al., 2018) and 2,5-dimethylpyrazine was reported to show strong antifungal activity against the grapevine pathogenic fungus *Phaeomoniella chlamydospora* (Haidar et al., 2016). The antifungal effect of 2-methyl-1-butanol and 3-methyl-1-butanol was also reported in several studies (Ando et al., 2012; Raza et al., 2015; Phoka et al., 2020). Therefore, different fungi may have varied reactions to the same volatile compound produced by bacteria.

Our results of antifungal bacterial volatiles may explain results from a previous study by Tenuta et al. (2002) about the reduction of wilt caused by *V. dahliae* after application of liquid swine manure to a potato field due to volatile fatty acids (VFAs) contained therein (mainly acetic, propionic, and isobutyric acids). The inhibition of the germination of microsclerotia was reversed when microsclerotia were transferred to VFA free medium after being exposed to sub-lethal amounts of VFAs.

## Conclusion

In the present study, we investigated the effect of soil microorganisms and other factors such as soil moisture and nutrients on microsclerotia dormancy and germination. The results showed that absence of external carbon source is not a crucial factor to keep microsclerotia in dormancy and suggest a potential inhibitory effect of soil bacteria on microsclerotia germination. Furthermore, an antifungal effect of VOCs produced by soil bacteria on the germination of microsclerotia was discovered and VOCs with an inhibitory potential against *V. longisporum* were identified. Our study provides new insights into the factors influencing microsclerotia dormancy and germination in the soil. However, in order to fully understand the mechanisms regulating dormancy and germination of microsclerotia, the impact of other soil living organisms and of host plant roots and their exudates need to be considered (Bakker et al., 2020; Trivedi et al., 2020). Such research can provide knowledge to develop innovative strategies for the control of diseases caused by Verticillium pathogens, either by enhancing dormancy in the presence of host plants or inducing germination of microsclerotia in the absence of their hosts.

## Data availability statement

The raw data supporting the conclusions of this article will be made available by the authors, without undue reservation.

## Author contributions

SS performed all the above mentioned experiments. SS, SR, and AT contributed to the experimental design and manuscript writing. AT, SR, and YW provided scientific advice in the study. All authors contributed to the article and approved the submitted version.

## Funding

This work was funded by the Department of Crop Sciences, Georg-August-University Göttingen.

## Conflict of interest

The authors declare that the research was conducted in the absence of any commercial or financial relationships that could be construed as a potential conflict of interest.

## Publisher’s note

All claims expressed in this article are solely those of the authors and do not necessarily represent those of their affiliated organizations, or those of the publisher, the editors and the reviewers. Any product that may be evaluated in this article, or claim that may be made by its manufacturer, is not guaranteed or endorsed by the publisher.
