# Supplementary material for: Dormancy and germination of microsclerotia of Verticillium longisporum are regulated by soil bacteria and soil moisture levels but not by nutrients
Source: Front Microbiol. 2022 Sep 23;13:979218. doi: 10.3389/fmicb.2022.979218 (PMC9539216; doi:10.3389/fmicb.2022.979218)
Supplement: Supplementary file 1 [file Data_Sheet_1.zip › Supplementary Figures Captions.pdf]

Supplementary Figure S1 Effects of water and nutrients on germination rate of microsclerotia at room temperature under sterile conditions *in vitro*. G0.2, G2 and PDB indicates 0.2%, 2% glucose solution and Potato Dextrose Broth, respectively. Error bars indicate standard error of the mean (n=3) (ANOVA test).

Supplementary Figure S2 Germination rate of microsclerotia after 2 days of incubation in sterile and unsterile grassland soil at different soil humidity levels. MWHC indicates maximal water holding capacity. *** indicates a statistically significant difference at $P < 0.001$ between different soil treatments at the same humidity level (ANOVA test). Different letters indicate significant differences at $p < 0.05$ between different humidity levels of same soil treatment. Error bars indicate standard error of the mean (n=3) (Tukey's test).

Supplementary Figure S3 Effect of different concentrations of *E. coli* on the germination rate of microsclerotia of *V. longisporum* after 24 hours of incubation. Undiluted means stock suspension of bacteria without dilution (5 x 108 CFU/mL). Numbers on x-axis indicate 2-, 10-, 16- and 100-fold dilution of stock suspension. Different letters indicate significant differences ($p < 0.05$) between treatments. Error bars indicate standard error of the mean (n=3) (Tukey's test).

Supplementary Figure S4 Effect of dead bacteria, bacterial culture filtrate and living bacteria on microsclerotia germination, (A) *Escherichia coli*, (B) soil bacteria 2. Different letters indicate significant differences ($p < 0.05$) between different treatments 6 and 24 hours after treatment, respectively. Error bars indicate standard error of the mean (n=4) (Pairwise Wilcox test).

Supplementary Figure S5 Effects of bacterial volatiles on the growth of colonies originating from microsclerotia after 7 days of incubation. (A) *Escherichia coli* and (B) soil bacteria 2. Undiluted means stock suspension of bacteria without dilution. Numbers on x-axis indicate 100-, 10,000- and 1,000,000-fold dilutions of stock suspension. Different letters indicate significant differences ($p < 0.05$) between treatments. Error bars indicate standard error of the mean (n=3) (Tukey's test).

Supplementary Figure S6 Plant height of oilseed rape inoculated with microsclerotia of *V. longisporum* grown in autoclaved and non-autoclaved soil. AC indicates control plants grown in autoclaved soil, AI indicates inoculated plants grown in autoclaved soil, NC indicates control plants grown in non-autoclaved soil and NI indicates inoculated plants grown in non-autoclaved soil. Different lowercase and uppercase letters indicate significant differences ($p < 0.05$) between treatments 7 and 10 weeks after treatment, respectively. Error bars indicate standard error of the mean (n=20) (Pairwise Wilcox test).
