# Supplementary figures and images for: Dormancy and germination of microsclerotia of Verticillium longisporum are regulated by soil bacteria and soil moisture levels but not by nutrients
Source: Front Microbiol. 2022 Sep 23;13:979218. doi: 10.3389/fmicb.2022.979218 (PMC9539216; doi:10.3389/fmicb.2022.979218)

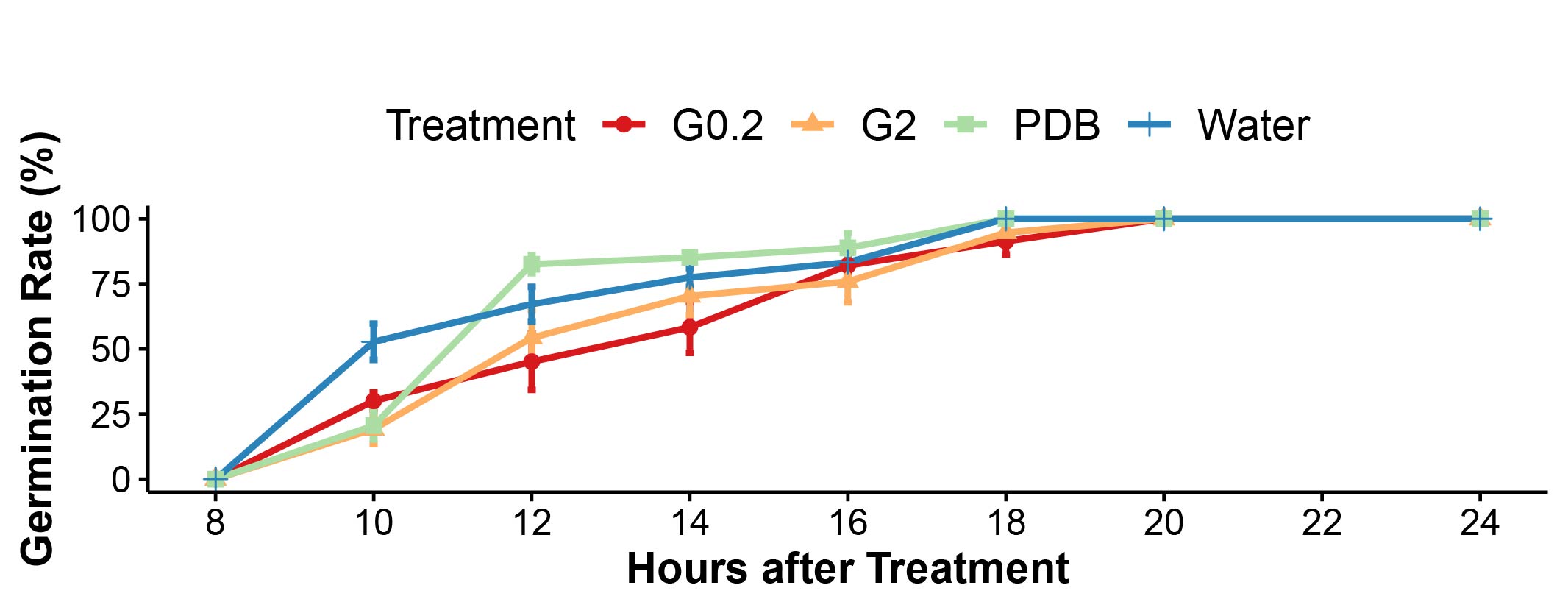

Supplement: Supplementary file 1 [file Data_Sheet_1.zip › Supplementary Figure S1.jpg]

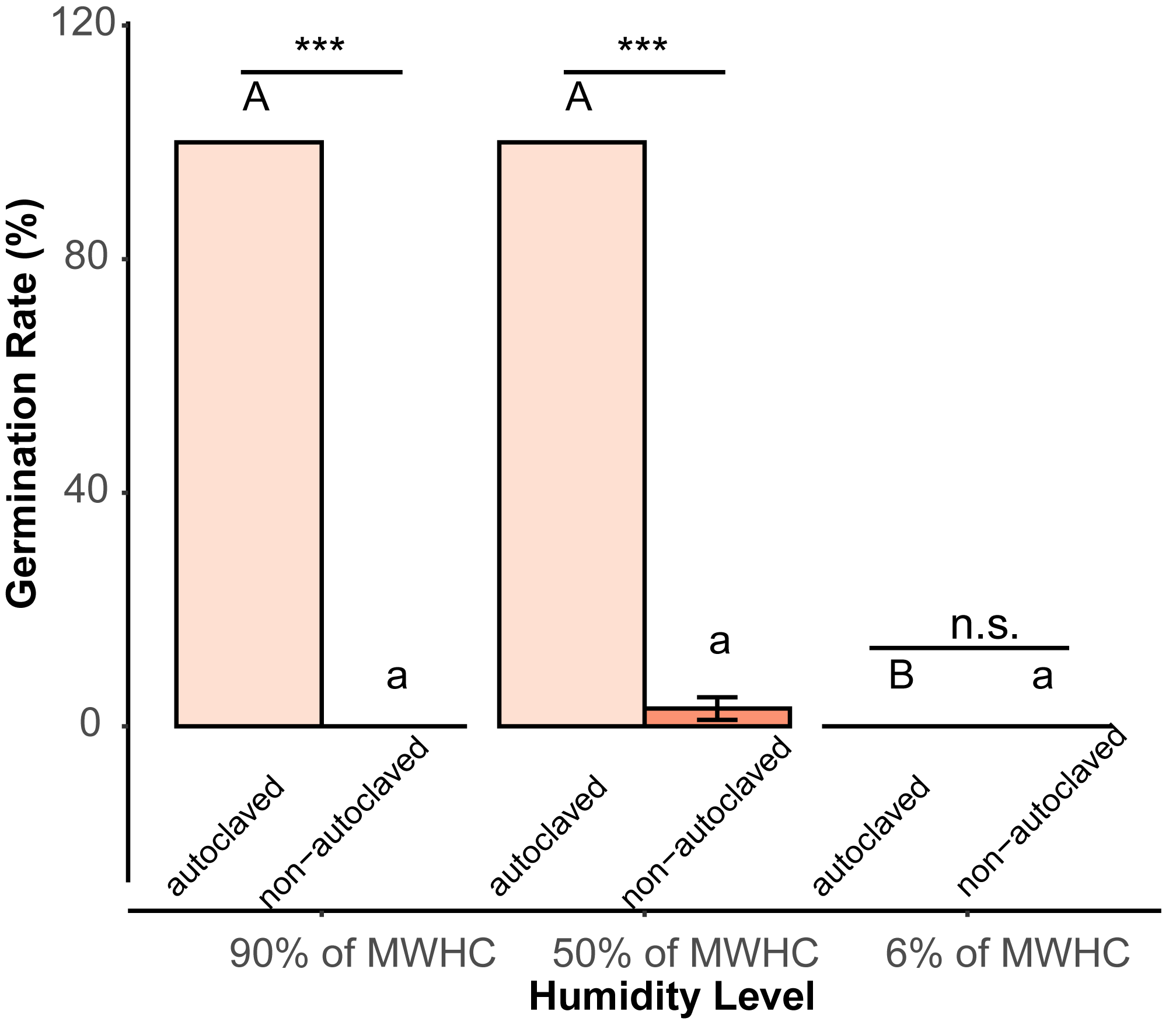

Supplement: Supplementary file 1 [file Data_Sheet_1.zip › Supplementary Figure S2.tif]

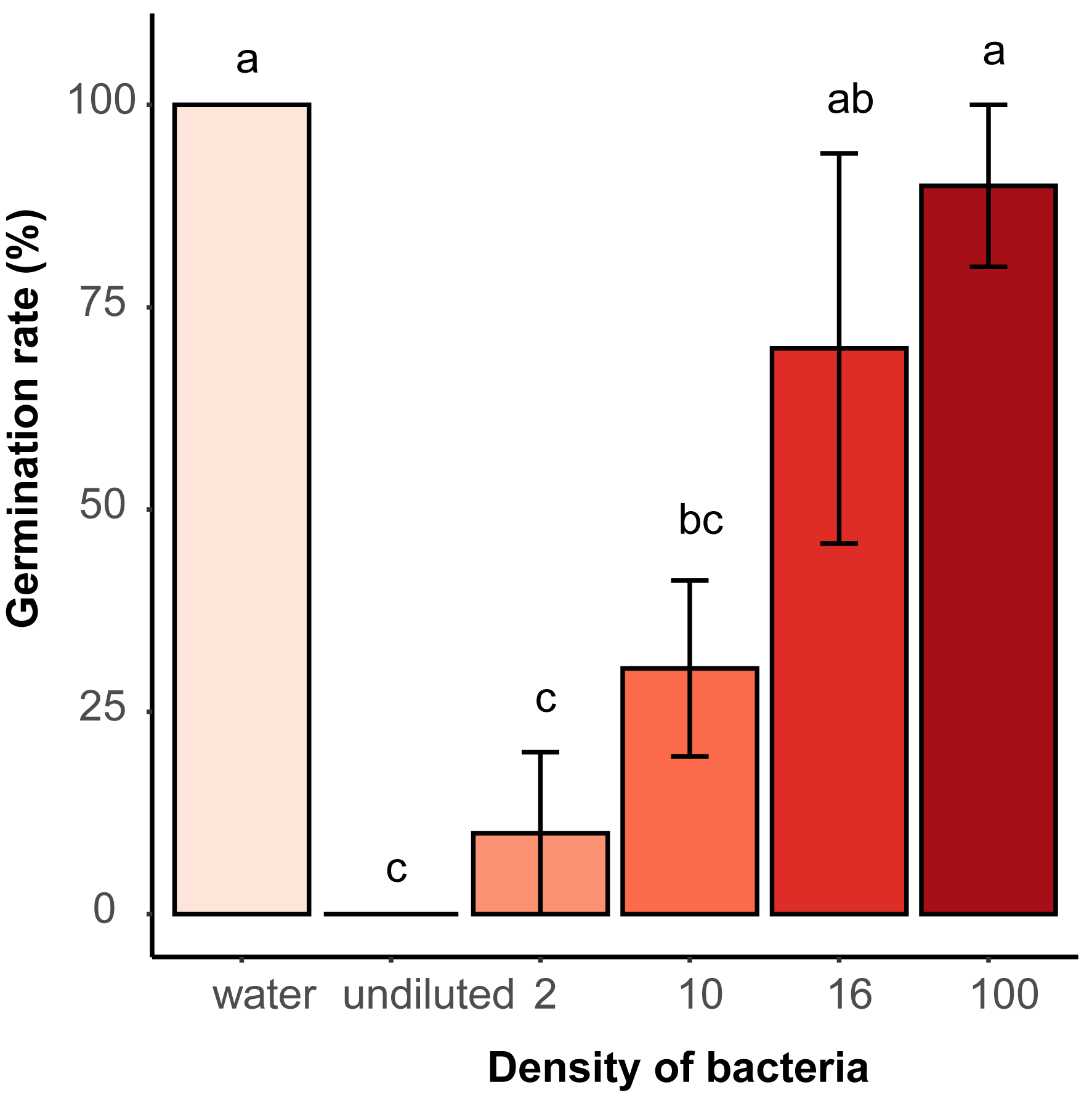

Supplement: Supplementary file 1 [file Data_Sheet_1.zip › Supplementary Figure S3.tif]

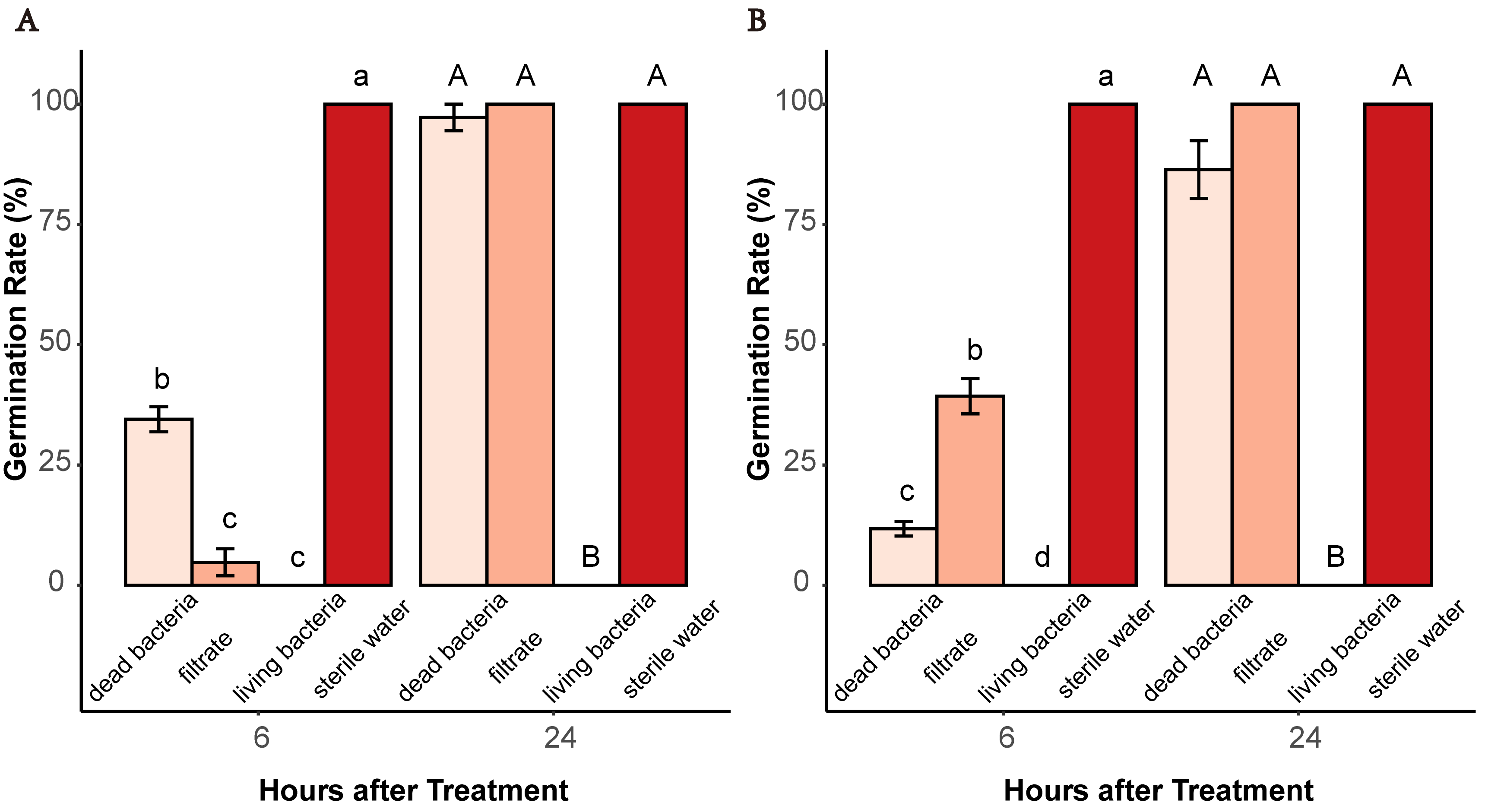

Supplement: Supplementary file 1 [file Data_Sheet_1.zip › Supplementary Figure S4.tif]

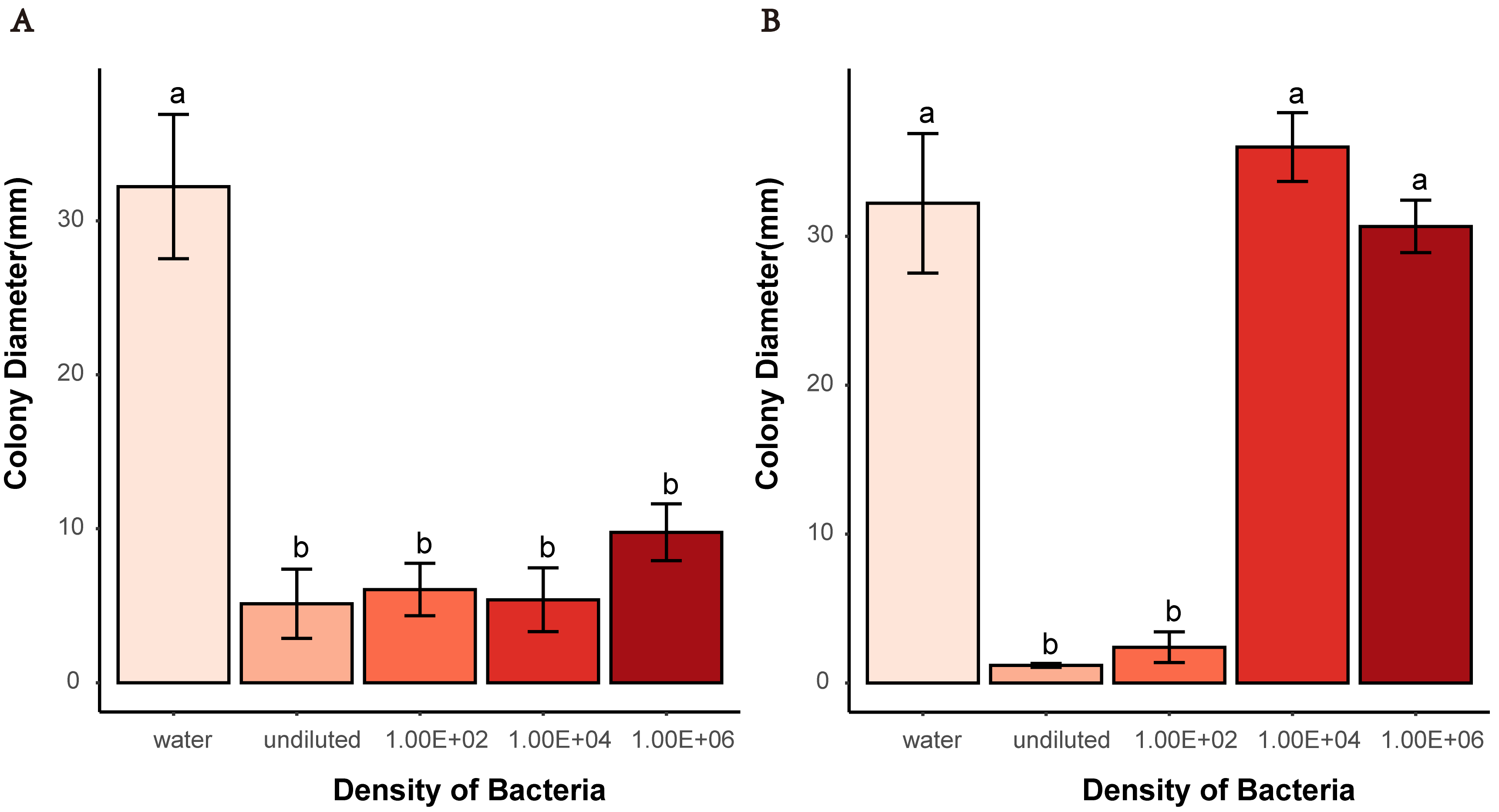

Supplement: Supplementary file 1 [file Data_Sheet_1.zip › Supplementary Figure S5.tif]

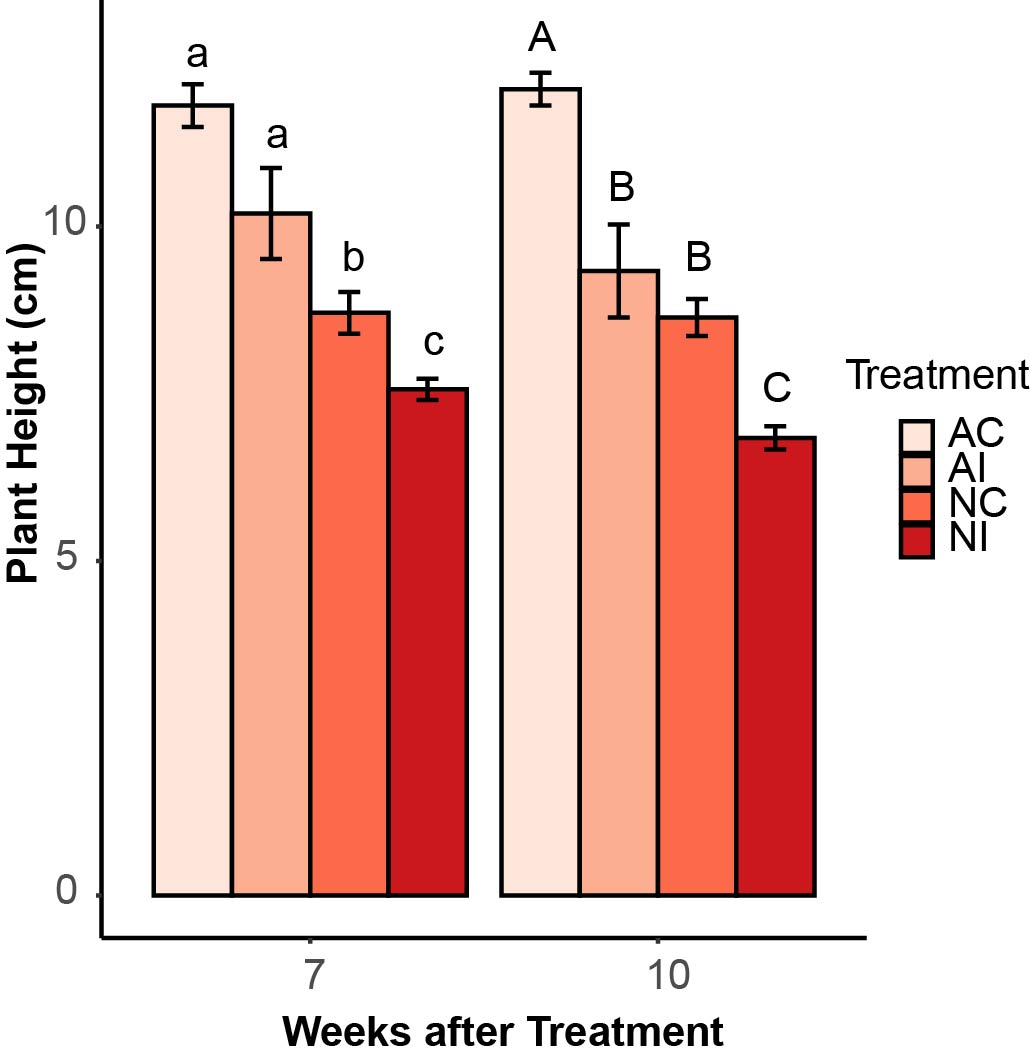

Supplement: Supplementary file 1 [file Data_Sheet_1.zip › Supplementary Figure S6.jpg]
